# Why Ebbinghaus’ savings method from 1885 is a very ‘pure’ measure of memory performance

**DOI:** 10.3758/s13423-022-02172-3

**Published:** 2022-09-07

**Authors:** Jaap M. J. Murre, Antonio G. Chessa

**Affiliations:** 1 Department of Psychology, University of Amsterdam, P.O. Box 15915, 1001 Amsterdam, NK Netherlands; 2 CBS – Statistics Netherlands, Department of Consumer Prices, P.O. Box 24500, 2490 The Hague, HA Netherlands

**Keywords:** Memory, Forgetting curve, Savings, Ebbinghaus

## Abstract

This paper analyzes the savings measures introduced by Ebbinghaus in his monograph of 1885. He measured memory retention in terms of the learning time saved in subsequent study trials relative to the time spent on the first learning trial. We prove mathematically that Ebbinghaus’ savings measure is independent of initial encoding strength, learning time, and relearning times. This theoretical model-free result demonstrates that savings is in a sense a very ‘pure’ measure of memory. Considering savings as an old-fashioned and unwieldy measure of memory may be unwarranted given this interesting property, which hitherto seems to have been overlooked. We contrast this with often used forgetting functions based on recall probability, such as the power function, showing that we should expect a lower forgetting rate in the initial portion of the curve for material that has been learned less well.

## Introduction

It is hard to overestimate the importance of Hermann Ebbinghaus’ contribution to experimental psychology (Ebbinghaus, 1880, 1885, 1913/1885). In 1885, he published a monograph with a series of rigorous experiments on the basis of his habilitation’s thesis from 1880 on the shape of learning and forgetting. He introduced the use of nonsense syllables, which had more uniform characteristics than words or other verbal material, though verified his findings with more natural material such as poems. He was, furthermore, one of the first psychologists to make extensive use of statistics and mathematical modeling, notably fitting mathematical equations to his now famous forgetting curve.

Ebbinghaus (1913; we shall mainly refer to this early English translation, which is readily available) based nearly all of his experiments on the *savings* measure of learning and memory, which is defined as the relative amount of time saved on the second learning trial as a result of having had the first. So, if it takes only half the time to relearn a list, savings will be 0.5 (we use proportions here instead of percentages). If it takes exactly as long to relearn the list as it took to learn it originally, then savings is 0. If the list is still completely known at the second trial (i.e., no forgetting at all), then savings is 1. If we call savings after time *t* as *Q*(*t*), this can be summarized as:

$$Q(t)=\frac{L-{L}_t}{L}.$$

Here, *L* is the time (or number of repetitions) needed to learn the material to criterion at *t* = 0 and *L*_*t*_ is the time needed to relearn the material at time *t*.

The savings measure of learning and forgetting remained popular for several decades (Ammons et al., 1958; Boreas, 1930; Finkenbinder, 1913; Krueger, 1929; Radossawljewitsch, 1907), but is used only sporadically today (see https://osf.io/xtfnd for a data repository with savings data and curve fits). This is remarkable because many papers use Ebbinghaus’ classic savings data to test hypothesis about the shape of learning and forgetting (e.g., Anderson & Schooler, 1991; Rubin & Wenzel, 1996; Wixted & Ebbesen, 1991). Nelson (1985) summarizes several reasons why the savings measure was abandoned. First, there may be a relatively large learning-to-learn effect, because it takes a lot of experience with the nonsense syllable materials and the savings procedure to get used to them. During this time, performance continues to improve, simply because the subject is still getting used to the experimental method. Ebbinghaus realized this, which is why he spent a relatively long time learning and relearning lists before he started the actual experiment; we followed him in this in our replication of his classic forgetting curve (Murre & Dros, 2015). Second, savings scores may be unreliable when learning to once or twice correct, which had already been observed by Luh (1922). With learning to once correct, learning proceeds until the trial on which all nonsense syllables can be produced correctly (100% correct); with learning to twice correct, learning proceeds until two successive trials are 100% correct. This can be remedied by learning to a lower criterion, such as 80% or 50% correct. Third, the savings measure can only be interpreted on an interval scale if the underlying learning process proceeds in a linear fashion with learning trials or time. If not, it is not really possible to compare different magnitudes of the savings measure (Nelson, 1985, p. 475). This is most troubling because if the underlying learning process is nonlinear and unknown, fitting a “forgetting curve” to savings data becomes meaningless (Wixted, 1990). As we shall see below, assuming a linear learning process for Ebbinghaus’ data seems warranted.

In this paper, we analyze the savings measure in more detail. Our analyses demonstrate that, unexpectedly, Ebbinghaus’ savings measure is an exceptionally good measure of memory, which in many ways is to be preferred above the more usual measures such as free or cued recall. In particular, we prove analytically that under many circumstances, savings is a “pure” retention measure: the shape of forgetting as measured through the savings method does not depend on the strength of the initial memory encoding or initial length of learning.

## Analysis of Ebbinghaus’ Savings Measures

Savings experiments differ from other memory retention experiments in the role of learning time. In recall experiments, subjects typically learn items for some pre-established time, during which memory encoding is hypothesized to take place. In Ebbinghaus’ classical savings experiment, learning time is a running variable where subjects continue learning until a pre-set criterion has been reached (e.g., one perfect recitation or 80% correct). One implication of the difference between the classical savings and other types of memory measures is that recall always (also) takes place immediately after a learning trial in a savings experiment. This is necessary to assess the initial level of learning. One might suspect that leaving learning time a free variable leads to less controlled testing, but as we argue here, the opposite is true: The savings measure may well suffer from fewer confounding variables than recall or recognition measures.

In our analysis, we first derive the expression for Ebbinghaus’ classical savings measure. Let *L* and *L*_*t*_ denote the learning times at the first and the second trial, respectively, which are separated by a retention lag *t*. Without loss of generalization, we assume that learning continues on both trials until the stimulus material can be fully recalled, rather than, say, to 80% correct. Learning, thus, continues until a certain “minimum memory strength” or “intensity” has been acquired that leads to successful recall. To make our line of reasoning easier to follow, we present two versions of our analyses, where the second one makes fewer assumptions than the first: (1) This analysis is based on a specific forgetting function and serves as an example for the next version. (2) Here, we show that the analysis of Version 1 can be generalized to all viable forgetting functions.

### Analysis Based on Power Function Decline

We assume that learning proceeds until the memory trace has reached a strong enough intensity to produce learned behavior that meets the criterion (e.g., perfect recall of a list of words or nonsense syllables). We often denote memory intensity as *μ* where 0 ≤ *μ* ≤ 1. Without further addressing the theoretical implications of this here, we note that this assumes that for the purposes of our analyses it is meaningful to speak of the scalar-valued “intensity” of a memory trace. Another assumption – which we shall pursue in more detail below – is that intensity increases linearly with learning time *L*. After a delay of *t* time units (e.g., seconds or days or learning trials), the relearning time to reach the set criterion once again is denoted as *L*_*t*_. We assume for Version 1 of the proof that the original strength has declined with a power function to *μ*(1 + *t*)^−*a*^, where *a* ≥ 0 is the forgetting parameter. During the relearning trial, the declined strength is increased through additional learning during *L*_*t*_ seconds, giving an additional strength of *νL*_*t*_, denoting the learning rate as *ν*. This is a formal introduction of the linear learning assumption. Keeping in mind that *μ* = *νL*,we now have:

$$\nu L=\mu {\left(1+t\right)}^{-a}+\nu {L}_t=\nu L{\left(1+t\right)}^{-a}+\nu {L}_t=\nu \left[L{\left(1+t\right)}^{-a}+{L}_t\right]$$

or

$$L=L{\left(1+t\right)}^{-a}+{L}_t$$

We can rearrange this as:

$$\frac{L-{L}_t}{L}={\left(1+t\right)}^{-a}.$$

But this is the expression for the savings measure *Q*(*t*), so we have:

$$Q(t)=\frac{L-{L}_t}{L}={\left(1+t\right)}^{-a}.$$

In other words, if we assume power function decline of the underlying strength of a memory trace, the savings method will measure exactly this function, independently of the original memory strength.

### Analysis Generalized to any Decline Function

The previous analysis can easily be generalized to any decline function f(*t*), assuming that the original strength of the memory trace has declined to *μ*f(*t*). We then have

$$\nu L=\mu \mathrm{f}(t)+\nu {L}_t=\nu L\mathrm{f}(t)+\nu {L}_t=\nu \left[L\mathrm{f}(t)+{L}_t\right]$$

or

$$L=L\mathrm{f}(t)+{L}_t,$$

giving

$$Q(t)=\frac{L-{L}_t}{L}=\mathrm{f}(t).$$

We can summarize this result as follows: If there is some function f(*t*) that describes the decline of the memory strength underlying memory performance as a function of time *t*, the savings method will directly measure this. Moreover, the savings method is completely independent of the initial learning strength and learning criterion: the observed savings-based forgetting curves should be the same for a criterion of 30%, 80%, or 100%. In this sense, the savings method is a “pure” measure of underlying memory strength.

#### Varying Initial Level of Learning

Ebbinghaus (1913, Ch. VI) also includes an experiment where he systematically varied the initial level of learning by increasing the number of initial learning trials on the first day. After 24 h, he relearned until once successful recall and measured the learning time on the second day. In a similar manner as that described above, we can derive the expression for the expected relation between learning time on Day 1 and Day 2, as follows.

Suppose it would take *L* seconds to learn a list to some criterion (e.g., once correct) corresponding with a memory intensity of *μ*. Now, instead of learning to criterion, we learn for fewer seconds, *L*_1_ < *L,* at Time 1. That is, we stop learning *before* we have reached the criterion. Then at Time 2, which takes place *t* seconds later (in Ebbinghaus’ case, 24 h later), we *do* learn until the criterion has been reached, this time taking *L*_2_ seconds.

Assuming a linear learning process and an initial learning trial of *L*_1_ seconds, this gives an initial intensity after learning on Day 1 of *μ*_1_ = *νL*_1_, where *ν* is the learning rate. After *t* seconds have passed, the intensity will have declined as described by the forgetting function. Above, we found the forgetting function to be equivalent to the savings measure itself, *Q*(*t*). So, on Day 2 after *t* seconds have passed, we retain an intensity of *νL*_1_*Q*(*t*). This intensity must now be increased to reach the memory intensity *μ* corresponding to the criterion by doing additional learning trials for *L*_2_ seconds. This gives an extra contribution to the intensity of *νL*_2_. We are interested in how *L*_2_ depends on *L*_1_.

We have:

$$\nu {L}_1Q(t)+\nu {L}_2=\mu =\nu L.$$

From this, we can derive the relationship between partial learning time *L*_1_ and relearning time to criterion *L*_2_, where savings at time *t* is a *non-free parameter*:

$${L}_2=L-Q(t){L}_1.$$

This relationship is a simple linear one and we are able to predict this learning data without using any estimated parameters, as is shown below.

Table 1 summarizes the mean relearning time as a function of the number of initial learning trials with only relearning until successful recall, as reported by Ebbinghaus (1913, Ch. VI). In the first column of this table, we see that the mean relearning time after zero initial learning trials is equal to 1,270 s, which we use as an estimate for *L*. Because the stimulus material used in this experiment is the same as the lists used in Ebbinghaus’ classical savings experiment, for the 24-h data point, Ebbinghaus reports that *Q*(*t*) = 0.337, where *t* = 24 h after initial learning.

**Table 1:** Relearning time in seconds until once correct recall on Day 2 as a function of a fixed number of initial learning trials on Day 1 (Ebbinghaus, 1913, Ch. VI)

Number of initial trials	0	8	16	24	32	42	53	64
Mean relearning time, s	1,270	1,167	1,078	975	863	697	585	454

Ebbinghaus (1913, Ch. VI, p. 57) also reports that a repetition of a single 16-syllable series takes between 6.6 and 6.8 s. If we use an approximation of 6.7 s per list and noting there are six of such 16-syllable lists per repetition, then each repetition took about 40.2 s. The data and fit are shown in Fig. 2. The predicted function coincides well with the data points, explaining 99.75% of the variance (sum of squared differences is 5963.64). Note that this function was not fitted to the data but based on separate values reported by Ebbinghaus. If we allow a shorter time than 6.7 s per list, we find that 6.38 s explains the same amount of variance but gives the lowest attainable sum of squared differences, namely 1,521.49. Given the excellent fit, one might argue that the assumption of a linearly increasing intensity with time is a reasonable one for Ebbinghaus’ data.

## Discussion

**Fig. 1:**
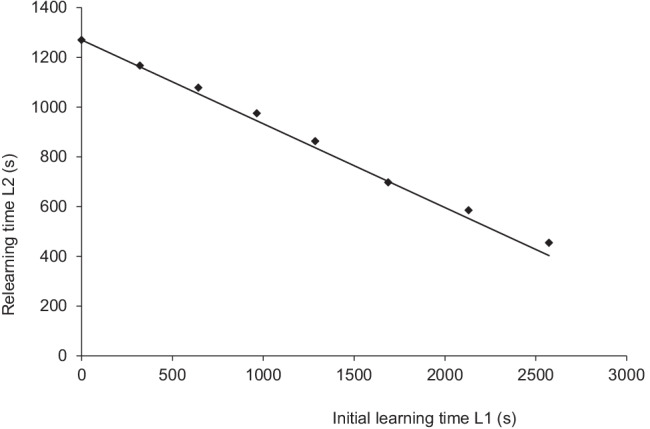
Varied levels of initial learning time *L*_1_ versus relearning time *L*_2_ to criterion after 24 h (Ebbinghaus, 1913, Ch. VI). The data are shown as diamonds. The predicted data are shown as a solid line

As we show above, Ebbinghaus’ classical savings function is independent of initial learning time and encoding strength and directly measures the underlying forgetting curve, assuming a learning process by which the memory intensity increases linearly with learning time. The fit of Ebbinghaus’ data relating initial learning time to relearning time in Fig. 1 further illustrates this, explaining nearly 100% of the variance without any free parameters.

**Fig. 2:**
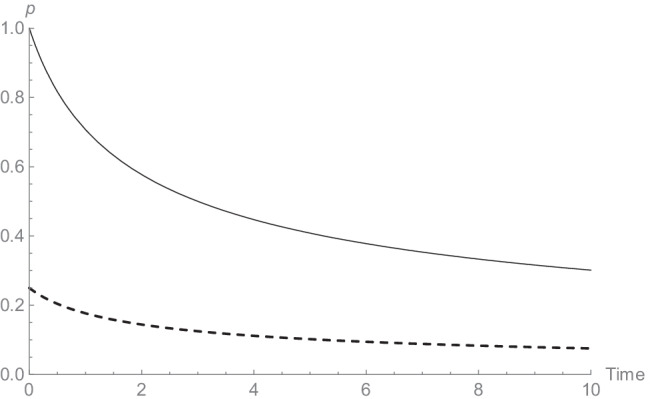
Power functions with *a* = 0.5 and two levels of initial intensity: *μ* = 1 (solid line) and *μ* = 0.25 (dashed line)

We should, perhaps, point out here that other measures of memory do not have this characteristic. For example, consider the power function, using probability of recall as a measure of memory: *p*(*t*) = *μ*(1 + *t*),^−*a*^ where *μ* is the initial intensity of the underlying learning process, which again is assumed to increase with learning time. If we take the first derivative of the forgetting function, we obtain the predicted initial forgetting rate for various levels of initial learning*μ*: *p*^′^(*t*) =  − *a*(1 + *t*)^−1 − *a*^*μ*. With various levels of initial learning at *t* = 0, we have *p*^′^(0) =  − *aμ*. In other words, if there is a stronger initial memory (with higher *μ*), there will be relatively higher forgetting rates at *t* = 0 (see Fig. 2 for an illustration).

It can easily be shown that the same result obtains for other forms of the power function, or for the exponential function (Loftus, 1985). More generally, any forgetting function of shape *p*(*t*) = *μ*f(*t*), where f(*t*) is a function that does not itself depend on *μ*, will give the same result because of the standard “constant factor rule” for finding the derivative of a product of a constant and a function: *p*^′^(*t*) = f^′^(*t*)*μ*.If f(t) is a declining function, at *t* = 0, f^′^(0) =  − *a*, for some positive constant *a*, so*p*^′^(*t*) =  − *aμ*. This means that for a large class of functions, we predict a lower forgetting rate in the initial portion of the curve for material that has been learned less well. This is also intuitively understandable from the idea that the role of the *μ* parameter is to shrink (or stretch, up to probability 1) the entire curve along the vertical axis; shrinking implies flattening, implying in turn lower forgetting rates. Of course, it will depend on the exact nature of a particular forgetting function f(*t*) how difficult it is to disentangle the effects of intensity (e.g., Wixted & Ebbesen, 1991). Our analysis of the relationship between learning and forgetting here is brief and incomplete. Indeed, there is a rich literature on this topic (Kauffman & Carlsen, 1989; Loftus, 1985; Slamecka & McElree, 1983; Yang et al., 2016) with ongoing experimentation and theorizing (Fisher & Radvansky, 2019; Radvansky et al., 2022; Rivera-Lares et al., 2022). We merely intend to illustrate that the savings measure cannot directly be compared with other measures of memory – for example, probability correct in cued recall – but that from a theoretical perspective it is expected to behave differently. In particular, from a theoretical perspective, its shape is independent of level of initial learning.

In conclusion, the savings measure introduced by Ebbinghaus (Ebbinghaus, 1880, 1885, 1913/1885) should not be regarded as old-fashioned and unwieldy. Among all memory retention measures proposed, it may be the purest one and worthy of renewed attention.
